# Folgen der COVID-19-Krise auf die kurz-, mittel- und langfristigen Beschäftigungs- und Entlohnungschancen nach Berufen

**DOI:** 10.1007/s11943-021-00284-1

**Published:** 2021-06-21

**Authors:** Tobias Maier, Anke Mönnig, Gerd Zika

**Affiliations:** 1 Bundesinstitut für Berufsbildung (BIBB), Bonn, Deutschland; 2 Gesellschaft für Wirtschaftliche Strukturforschung mbH (GWS), Osnabrück, Deutschland; 3 Institut für Arbeitsmarkt- und Berufsforschung (IAB) der Bundesagentur für Arbeit (BA), Nürnberg, Deutschland

**Keywords:** Projektion, Berufsstruktur, Lohnentwicklung, COVID-19-Pandemie, Szenarioanalyse, Input-Output, Arbeitsmarkt, Projection, Occupational structure, Wage development, COVID-19 pandemic, Scenario analysis, Input-output, Labour market

## Abstract

Dieser Beitrag stellt das Modellsystem QINFORGE vor. Es ist durch die Berücksichtigung beruflicher Mobilitätsprozesse in der Lage, Anpassungsreaktionen zwischen Arbeitsangebot und -bedarf auf beruflicher Ebene in der kurzen und langen Frist aufzuzeigen. Das Modell wird genutzt, um die Auswirkungen der COVID-19-Krise auf die Beschäftigungs- und Entlohnungschancen in Berufen zu verdeutlichen. Hierfür werden zwei Szenarien miteinander vergleichen: Ein „kontrafaktisches Szenario“ schreibt langfristige Verhaltensweisen und Trends ausgehend vom Jahr 2019 fort. Die „Basisprojektion“ versucht hingegen, die Konjunktur in der kurzen Frist auf Branchenebene mit abzubilden. Hierdurch werden modellendogen Verhaltensänderungen auf Berufsebene angeregt.

Die Krise wird die Wirtschaftsleistung Deutschlands langfristig um drei Jahre zurückwerfen. Da sich aufgrund der schlechteren Arbeitsmarktlage aber auch einige Erwerbspersonen, insbesondere Frauen, vom Arbeitsmarkt zurückziehen werden, steigt die prognostizierte Zahl der Erwerbslosen in den Jahren 2020 und 2021 in der Basisprojektion nur um rund 400.000 Personen im Vergleich zum kontrafaktischen Szenario an. Langfristig stellt das knappe Arbeitsangebot hingegen einen hemmenden Faktor für das Wirtschaftswachstum dar. Zudem wird durch die Krise in der kurzen und mittleren Frist das gesamtwirtschaftliche Lohnwachstum gehemmt.

Die Ergebnisse des Modells zeigen, dass durch die Krise vor allem Berufe in der Gastronomie, dem Tourismus, sowie in Kunst und Kultur betroffen sind. Ab dem Jahr 2025 wird hier jedoch wieder das Lohn- und Beschäftigungsniveau erreicht, das auch im kontrafaktischen Szenario erreicht worden wäre. Bei den kurzfristigen Lohnsteigerungen in den Gesundheitsberufen handelt es sich hingegen um Vorzieheffekte. Auf lange Sicht ist in der Basisprojektion gegenüber dem „kontrafaktischen Szenario“ kein höheres Lohnniveau zu erwarten. Das Arbeitsangebot in den Gesundheitsberufen – insbesondere in der Pflege – könnte durch die Krise zwar zunehmen, dies wäre jedoch darauf zurückzuführen, dass die Erwerbschancen und Entlohnungsmöglichkeiten außerhalb der Pflegeberufe durch die COVID-19-Pandemie vergleichsweise geringer werden könnten.

## Abstract (en)

This paper presents the model system QINFORGE. By taking into account occupational mobility matrices, the model is able to show adaptation reactions between labour supply and demand at the occupational level in the short- and long-term. We use the model to illustrate the effects of the COVID-19 crisis on employment and remuneration opportunities in occupations. For this purpose, we compare two scenarios. On the one hand, a “counterfactual scenario” extrapolates long-term behaviour and trends starting from the year 2019. The “baseline projection”, on the other hand, models the economic situation in the short term at the industry level. This stimulates model-based behavioural changes at the occupational level.

The crisis will set back Germany’s economic performance by three years in the long-term. As the worsening labour market situation will also cause lower labour participation rates, especially among women, the number of the projected unemployed in the “baseline projection” for 2020 and 2021 will only rise by around 400,000 compared with the “counterfactual scenario”. In the long term, however, the scarce supply of labour will be an inhibiting factor for economic growth. In addition, the crisis will inhibit overall wage growth in the short and medium term

The results of the model show that occupations in gastronomy, tourism, and art and culture are particularly affected by the crisis. From 2025, however, the wage and employment levels that would have been achieved in the “counterfactual scenario” will be reached. By contrast, the short-term wage increases in the health care occupations are anticipatory effects. In the long-term, the baseline-projection does not forecast higher wage levels than the “counterfactual scenario”. Nevertheless, the labour supply in the health care occupations, especially in nursing, could increase in the long term because of the crisis. However, this will be because the COVID 19 pandemic may reduce the employment and wage opportunities that are available to health care professionals outside of the health care sector.

## Einleitung

Für die Bildungs- und Arbeitsmarktpolitik ist es relevant zu wissen, welche Art von Tätigkeiten für die Herstellung von Gütern und Erbringung von Dienstleistungen auf dem Arbeitsmarkt nachgefragt werden und inwieweit die dem Arbeitsmarkt zur Verfügung stehenden Arbeitskräfte diese Tätigkeiten ausüben können. Denn Passungsprobleme, sowohl auf beruflicher als auch auf regionaler Ebene, verursachen in der Regel volkswirtschaftliche Kosten, da Arbeitslose beispielsweise Anspruch auf Sozial- und zum Teil auch auf Versicherungsleistungen haben oder Wertschöpfung verloren geht. Für Individuen haben mögliche Erwerbs- und Entlohnungschancen hingegen unmittelbare und langfristige Konsequenzen auf ihre finanziellen Ressourcen. Wirtschaftliche Krisen, wie durch die COVID-19(Coronavirus SARS-CoV-2)-Pandemie ausgelöst, können diese Erwerbs- und Entlohnungschancen gefährden. Es bedarf deshalb der Analyse, wie die COVID-19-Pandemie auf berufsspezifische Arbeitsmärkte und Entlohnungschancen einwirken könnte. Ein Blick auf den Arbeitsmarkt der Zukunft kann helfen, mögliche Problemfelder bereits frühzeitig zu erkennen und entsprechende Handlungsalternativen zu erarbeiten. Obwohl somit ein begründetes Interesse am Arbeitsmarkt der Zukunft besteht, werden praktische Annäherungsversuche, diesen zu beschreiben, kritisch betrachtet.

Die Kritik an Projektionen wird zumeist über ihre Praxisbewährung formuliert. Schließlich sei eine Vielzahl vergangener Prognosen bislang nicht eingetroffen bzw. die mit ihnen verbundenen Erwartungen nicht bestätigt. Dieser Erwartungsenttäuschung geht das Verständnis voraus, dass eine Prognose unter vollständiger Kenntnis der Entwicklung aller für die Prognose relevanten Parametern zustande kommt (Lucas 1976). Tatsächlich sind längerfristige Prognosen – egal für welche Fragestellung – jedoch immer bedingte Prognosen, deren Geltungsbereich im Sinne eines Wenn-Dann-Charakters nur innerhalb definierter Rahmenbedingungen gilt (Helmrich und Zika 2019).

Zur Lösung der Unsicherheitsthematik hat sich bei Projektionen deshalb die Szenarienanalyse (Helmrich et al. 2013) etabliert. Bei einer transparenten Nennung der Prognoseannahmen werden einzelne Parameter der Prognose verändert, um deren langfristige Auswirkungen auf weitere Größen sichtbar zu machen. Zudem ist es ratsam, die Berechnungen regelmäßig zu erneuern. Das etwaige Fortbestehen von vormals identifizierten Verhaltensweisen muss regelmäßig überprüft sowie mögliche Auswirkungen kurzfristiger Ereignisse quantifiziert werden. Die derzeitige COVID-19-Pandemie stellt beispielsweise ein solch einschneidendes Ereignis dar, dessen kurz-, mittel- und langfristigen Auswirkungen auf den Arbeitsmarkt derzeit unbekannt sind.

Mit diesem Beitrag wird das Modellsystem QINFORGE vorgestellt. Es stellt den makroökonometrischen Kern der Qualifikations- und Berufsprojektionen (QuBe-Projekt) dar. Es lässt eine Abschätzung der möglichen mittel- und langfristigen Auswirkungen der COVID-19-Krise zu, indem die Krise selbst als eine Art „externer Schock“ interpretiert wird. So lassen sich über Veränderungen in der kurzen Frist auch persistente Folgen für die Erwerbs- und Einkommenschancen nach Berufen nachzeichnen. Abschn. 2 erläutert zunächst das Modellsystem. Um die kurz-, mittel- und langfristigen Auswirkungen der COVID-19-Krise darzustellen, wird eine „Basisprojektion“, welche die derzeitig bekannten Einschnitte in der wirtschaftlichen Entwicklung abbildet, mit einem „kontrafaktischen Szenario“ verglichen. Das kontrafaktische Szenario simuliert ausgehend vom Jahr 2019 eine Welt ohne Corona-Pandemie (Abschn. 3). Abschn. 4 zeigt die Unterschiede in den Entlohnungs- und Erwerbschancen nach Beruf, die sich in beiden Szenarien ergeben. Der Beitrag schließt in Abschn. 5 mit einem Fazit.

## Das Modellsystem QINFORGE

Die Langfristprojektionen des QuBe-Projektes bestehen aus einer Bevölkerungsprojektion (Fuchs et al. 2017), einem darauf aufsetzenden Bildungsmodell (Kalinowski et al. 2021) und dem makroökonometrischen Input-Output-Modell INFORGE (**In**terindustry **For**ecasting **Ge**rmany) (Ahlert et al. 2009; Maier et al. 2015). Alle drei Modelltypen gehen in das Modellsystem mit Namen QINFORGE ein. Die Module sind miteinander verknüpft, indem sie entweder exogenen Input für die Bestimmung von Teilgrößen in anderen Modulen liefern (wie beispielsweise die Bevölkerungsprojektion an die Bestimmung der Arbeitsnachfrage oder Arbeitsangebot) oder endogene Rückkopplungseffekte auf die Einzelmodule ausüben (wie beispielsweise gleichzeitig der Lohn das Arbeitsangebot bestimmt und das Arbeitsangebot auch die Lohnentwicklung beeinflusst). Die aus QINFORGE entstehenden Projektionen reichen bis in das Jahr 2040 und wurden bislang vorwiegend für die Untersuchung unterschiedlicher Fragestellungen zum Strukturwandel herangezogen (Mönnig et al. 2019, 2020, 2018; Zika et al. 2017, 2019).

Das Modellsystem QINFORGE hebt sich von den internationalen und nationalen Projektionsmodellen (Bakens et al. 2018; Bonin et al. 2007; Bundesamt für Statistik 2019; Bureau of Labor Statistics 2018; Cedefop 2018; France Stratégie und Dares 2018; Statistics Sweden 2017; vbw 2019; Wilson et al. 2016) ab, weil es die Kopplungsfunktion des Berufs zwischen der Arbeitsangebots- und -nachfrageseite explizit in das Zentrum der Analyse stellt. Dies ist möglich, da der erlernte Beruf der Befragten im Mikrozensus über den höchsten beruflichen Abschluss im Zusammenhang mit der Hauptfachrichtung in die nationale Klassifikation der Berufe 2010 (KldB 2010) überführt wurde (Maier und Helmrich 2012). Hierdurch können berufliche Mobilitätmatrizen zwischen erlerntem und ausgeübtem Beruf ausgewiesen und für die Gegenüberstellung des berufsspezifischen Arbeitsangebots mit dem Bedarf an Erwerbstätigen im Beruf genutzt werden. Dies ermöglicht die Identifikation von Arbeitskräfteknappheiten auf Berufsebene, welche wiederum Rückwirkungen auf die Bereitstellung von Waren und Dienstleistungen auf Branchenebene zeigen.

Der folgende Abschnitt zeigt zunächst die modulare Struktur von QINFORGE auf, um anschließend die einzelnen, für die vorliegende Fragestellung relevanten Modellelemente zu beschreiben.

### Modulare Struktur QINFORGE

**Figure 1:**
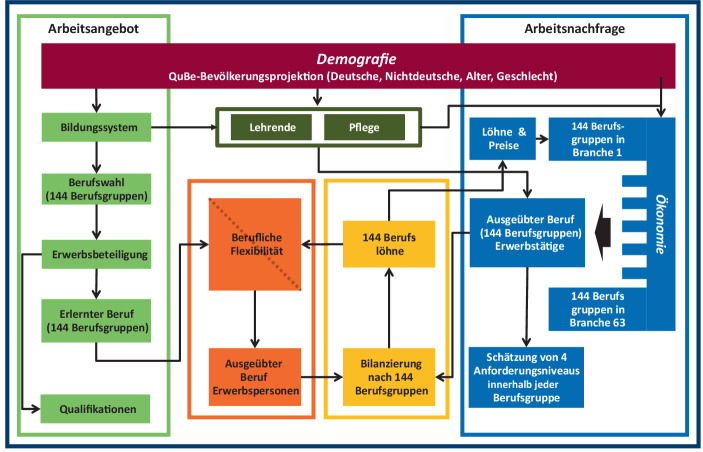

Abb. 1 gibt einen Überblick über die modulare Struktur von QINFORGE. Die demografische Entwicklung nach Alter, Geschlecht und Nationalität (roter Kasten in Abb. 1) beeinflusst sowohl das Arbeitsangebot als auch die Arbeitsnachfrage. Die Arbeitsnachfrage wird von der Bevölkerung über die Wirtschaftsentwicklung beeinflusst, wobei insbesondere die private Konsumnachfrage eine entscheidende Rolle spielt. Diese wird durch die Bevölkerung sowohl in ihrer Entwicklung (Sonnenburg et al. 2015) als auch in ihrer Struktur (Stöver und Wolter 2015; Aigner-Walder und Döring 2014) verändert. Darüber hinaus gehen mit einem sinkenden Erwerbspersonenpotenzial – ceteris paribus – die Produktionsmöglichkeiten in einer Volkswirtschaft zurück (Deutsche Bundesbank 2017). Da vor allem Pflege- und Erziehungsleistungen aufgrund ihrer Nachfrager eng mit der Bevölkerungsentwicklung verknüpft sind, wird die Bestimmung der Arbeitsnachfrage für diese beiden Wirtschaftszweige aus dem ökonomischen Modellkontext von INFORGE ausgegliedert und in zwei Sub-Module ausgelagert. Das Angebot an Fachkräften mit einem erlernten Beruf bestimmt sich aus der alters-, geschlechts- und nationalitätsspezifischen Berufswahl und der damit in Verbindung stehenden Erwerbsneigung (grüner Kasten in Abb. 1). Diese Erwerbsneigung wird von konjunkturellen Entwicklungen beeinflusst (Cedefop 2010; Kriechel et al. 2013).

Das Angebot an Personen nach erlerntem Beruf wird über im Mikrozensus ermittelte berufliche Flexibilitätsmatrizen in Erwerbspersonen für einen Beruf umgerechnet (oranger Kasten). Das makroökonometrische Input-Output-Modell INFORGE berechnet hierbei die Arbeitsnachfrage (blauer Kasten). Die Erwerbstätigen nach Beruf werden den berufsspezifischen Erwerbspersonen gegenübergestellt. Bei berufsspezifischen Knappheiten ergeben sich Veränderungen in der berufsspezifischen Lohnstruktur (gelber Kasten).

### Das Regressionsmodell INFORGE

Die Wahl des „richtigen“ Modelltyps hängt von der Fragestellung ab. Für eine langfristige Arbeitsmarktprojektion mit dem Ziel einer zukünftigen Engpassanalyse auf Berufsebene ist ein dynamisches Modell mit Strukturelementen notwendig. Allein diese beiden Bedingungen schließen Partialmodelle (Peichl et al. 2010) aus. Auch ausgeschlossen sind jegliche statischen Modelltypen. Ebenso unvereinbar ist damit die Philosophie des Gleichgewichtsgedankens (Meagher et al. 2015), da gerade zukünftige Ungleichgewichte aufgedeckt werden sollen. Der Input-Output-Ansatz ist für den Strukturaspekt von entscheidendem Vorteil. Das makroökonometrische Input-Output-Modell INFORGE entspricht diesen Anforderungen.

INFORGE (Ahlert et al. 2009; Maier et al. 2015) beschreibt das ökonomische Verhalten unterschiedlicher Wirtschaftsakteure^1^. Interindustrielle Verflechtungen werden explizit behandelt, so wie auch die Veränderung über die Zeit. Die Konten der Volkswirtschaftlichen Gesamtrechnungen (VGR) sind sowohl von der Nachfrage- als auch von der Angebotsseite ausgeglichen. Die Wirtschaftsentwicklung wird „bottom-up“ auf Wirtschaftszweig und Güterebene beschrieben und das Bruttoinlandsprodukt bestimmt sich aus der Summe seiner Teile. Die dafür notwendige hohe Anzahl an Gleichungen wird simultan gelöst. Die Input-Output-Tabellen sind dabei vollständig in das Kontensystem der VGR implementiert. Die Einkommensentstehung, -verteilung und -verwendung ist ganzheitlich abgebildet.

Bei Input-Output-Modellen werden auf der Arbeitsnachfrageseite zur Bestimmung der Erwerbstätigen in den Branchen unter anderem multi-sektorale makroökonomische Modelle verwendet, die um eine Input-Output-Tabelle aufgebaut sind (Wilson und Lindley 2007; für Deutschland: Maier et al. 2015; Prognos 2013).^2^ Input-Output-Tabellen beschreiben in einer sehr komprimierten Form die ökonomische Verflechtung zwischen Produktionsbereichen. Dynamische Input-Output-Modelle (Lehr et al. 2014) nutzen eine limitationale Produktionsfunktion, mit der die Produktion $y$ über die Endnachfrage $x$ und den technischen Koeffizienten $\left(I-A\right)^{-1}$ bestimmt wird.

1 $$y=\left(I-A\right)^{-1}x$$

Dynamische Input-Output-Modelle wie INFORGE kombinieren das Instrument der Ökonometrie mit der auf Input-Output-Tabellen basierenden limitationalen Produktionsfunktion, um Anpassungsprozesse in der Wirtschaft abzubilden. Sie ähneln den dynamischen „computable general equilibrium“ (CGE)-Modellen (Meagher et al. 2015). Beide Modelltypen lösen zwar simultan und zeigen Pfadabhängigkeiten auf – außerdem fußen sie auf derselben Datenbasis und nicht-linearen Zielfunktionen – allerdings unterscheiden sie sich in der theoretischen Fundierung der Modelltypen. Makroökonometrische Input-Output-Modelle basieren in der Regel auf post-keynesianischen Eigenschaften, wie beispielsweise unfreiwillige Arbeitslosigkeit, Unsicherheit oder träge Preisbildung. Diese Modelltypen können nicht optimieren – anders als die CGE-Modelle, welche auf Gleichgewichtspositionen ausgerichtet sind (West 2006). Optimierungsmöglichkeiten können hierbei nur durch Simulationsrechnungen erzeugt werden (Pollitt et al. 2019).

Der mit dem Modell INFORGE berechnete Wachstumspfad beinhaltet den technologischen Wandel – allerdings nur insoweit, als er in der Vergangenheit empirisch beobachtbar war. Hierbei definieren die technischen Koeffizienten der Input-Output-Beziehungen den technologischen Wandel (Mönnig 2016). Inputkoeffizienten sind der Anteil des Vorleistungseinsatzes eines Produkts im Verhältnis zur Produktion eines anderen Produkts. Veränderte Inputstrukturen können als Veränderungen im Produktmix innerhalb einer bestimmten Produktgruppe interpretiert werden (Meyer et al. 1999; Richter 1991). Ein autonomer technischer Fortschritt (Klauder 1986) besteht außerdem darin, dass Änderungen der Inputkoeffizienten in INFORGE endogen mit einem Zeittrend geschätzt werden.

Während die Produktion neben den technischen Koeffizienten von der heimischen Endnachfrage bestimmt wird (siehe Gl. 1), hängt die Nachfrage unter anderem von Preisveränderungen ab. Diese wiederum sind das Resultat eines Stückkostenansatzes in den jeweiligen Produktionsbereichen. Damit sind sowohl nachfrage- als auch angebotsseitige Entwicklungen in der Bestimmung der Produktion eingebunden.

Das ökonomische Rechenmodell ist auf die Größen der VGR und den korrespondierenden Input-Output-Tabellen des Statistischen Bundesamts angewiesen. Die VGR gibt u. a. das Arbeitsvolumen und die Zahl der Erwerbstätigen nach 63 Wirtschaftszweigen nach der Wirtschaftszweiggliederung WZ 2008 vor (StBA 2008).

Nationale Prognosemodelle wie INFORGE müssen zwangsläufig mit einer Reihe an Annahmen arbeiten, da nicht alle Variablen endogen bestimmt werden können. Es handelt sich hierbei um Vorgaben für die Entwicklung des Wechselkurses Euro/Dollar, um die Weltmarktpreisentwicklung von Rohstoffen, den Refinanzierungszins der EZB, um die deutsche Exportnachfrage und um die Bevölkerungsentwicklung in Deutschland.

Wechselkurse und Zinssätze sind – vor allem in der langen Frist (Sarno et al. 2002) – in der Regel schwierig zu projizieren, weshalb es sich in makroökonomischen Prognosemodellen bewährt hat, diese Parameter im Prognosezeitraum auf dem zuletzt bekannten Wert konstant zu halten. Damit nehmen beide Parameter keinen Einfluss auf die Prognose. Dennoch ist es möglich, die Wirkung einer Variation dieser Parameter mittels einer Szenariorechnung zu ermitteln. Weltmarktpreisentwicklung für Rohstoffe wie Erdöl, Erdgas oder Erze basieren auf Vorgaben anderer Institute wie der Internationalen Energieagentur (IEA 2019), dem Internationalen Währungsfonds (IMF 2019) und der Organisation für wirtschaftliche Zusammenarbeit und Entwicklung (OECD und FAO 2019).

Aufgrund der Exportorientierung Deutschlands ist die Entwicklung und Struktur des Welthandels eine wichtige Einflussgröße für die Entwicklung der deutschen Wirtschaft. Da der Export durch die Importnachfrage der Handelspartner bestimmt wird, ist eine endogene Bestimmung in einem nationalen Modell nicht möglich. Die Exportnachfrage muss daher vorgegeben werden. In INFORGE leitet sie sich aus dem Welthandelsmodell TINFORGE (Mönnig und Wolter 2020) ab. Für die Projektion wird ein durchschnittlicher jährlicher Anstieg des Welthandels zwischen 2020 bis 2040 von 4,7 % angenommen. Die nominalen Exporte Deutschlands wachsen um ca. 2,9 %.

Darüber hinaus wird die durchschnittliche Jahresarbeitszeit exogen gesetzt. Langfristig zeigt sich hier ein Trend zurückgehender Jahresarbeitszeiten. Wird das Angebot an Arbeitskräften jedoch zu knapp, muss die durchschnittliche Jahresarbeitszeit geringfügig erhöht werden. In der Basisprojektion wird sie bis zum Jahr 2025 noch leicht sinken, um anschließend bis zum 2035 wieder das heutige Niveau zu erreichen.

### Die QuBe-Bevölkerungsprojektion

Der demografische Wandel ist eine entscheidende Einflussgröße auf die ökonomische Entwicklung. Die Bevölkerungsentwicklung wird aus der QuBe-Bevölkerungsprojektion (Fuchs et al. 2017; Kalinowski et al. 2021) entnommen. Spezifika des Bevölkerungsmoduls sind zum einen die Unterscheidung zwischen Deutschen und Nichtdeutschen und zum anderen die Schätzung der einzelnen Komponenten (Geburtenziffern, Überlebenswahrscheinlichkeiten, Zu- und Abwanderungen sowie Einbürgerungen) mit zeitreihenanalytischen Methoden sowie deren Fortschreibung für die Zukunft (Fuchs et al. 2017; Gorodetski et al. 2016). Die Bevölkerung beeinflusst in INFORGE durch ihre Anzahl und Zusammensetzung nach Altersgruppen die Konsumausgaben und -struktur der privaten Haushalte (Stöver und Wolter 2015), die Ausgaben des Staates und auch die Bauinvestitionen.

Tab. 1 stellt die wesentlichen Größen der Bevölkerungsprojektion dar. Nach den hohen Nettozuwanderungsgewinnen im Jahr 2015 von rund 1,14 Mio. Personen in Folge des Syrien-Konfliktes (Zika et al. 2017) ist die Nettozuwanderung in den Folgejahren wieder abgeklungen. Allerdings lag sie in den Jahren 2017 und 2018 mit jeweils rund 400.000 Personen immer noch deutlich über dem langfristigen Durchschnitt von rund 300.000 Personen seit dem Jahr 1991. Die monatliche Wanderungsstatistik des Statistischen Bundesamtes zeigt im ersten Halbjahr 2020 einen starken Rückgang in den Wanderungen, der vermutlich mit den europaweiten *Shutdowns* in Verbindung steht. Der geschätzte Wanderungssaldo in Höhe von rund 411.000 Personen im Jahr 2020 (siehe Tab. 1) ist deshalb vermutlich zu hoch. Sofern die entsprechenden Wanderungswünsche in den Folgemonaten/-jahren nachgeholt werden, hat diese Überschätzung keine bedeutenden langfristigen Auswirkungen auf die Bevölkerungsprojektion.

**Table 1:** 

Jahr		2015	2020	2025	2030	2035	2040
Gesamtbevölkerung in Mio. Personen		82,18	83,41	84,01	84,09	83,94	83,67
Nichtdeutsche Anteil am Gesamt		11 %	13 %	14 %	15 %	15 %	16 %
Zuzüge in Mio. Personen		2,14	1,40	1,28	1,18	1,16	1,14
Fortzüge in Mio. Personen		1,00	1,00	1,00	1,00	0,99	0,99
Saldo in Mio. Personen		1,14	0,41	0,27	0,18	0,17	0,15
Zusammengefasste Geburtenziffer	Deutsche Frauen	1,43	1,44	1,52	1,55	1,57	1,57
	Ausländerinnen	1,96	2,09	2,06	2,05	2,05	2,05
Lebenserwartung in Jahren bei Geburt	Weiblich	83,06	83,80	84,80	85,61	86,19	86,68
	Männlich	78,18	78,94	79,86	80,74	81,57	82,34
Medianalter in Jahren	Weiblich	47,19	47,48	47,06	47,24	47,63	48,06
	Männlich	44,31	43,98	43,97	44,29	44,87	44,90

Quelle: QuBe-Projekt, sechste Welle, Bevölkerungsfortschreibung des Statistischen Bundesamtes

In der langfristigen Projektion sinkt die Nettozuwanderung von ihrem hohen Niveau auf rund 150.000 Personen im Jahr 2040 ab. Insgesamt wird aufgrund durchgehend positiver Zuwanderungsgewinne bis zum Jahr 2030 ein Bevölkerungswachstum auf rund 84,1 Mio. erwartet. Bis zum Jahr 2040 geht die Bevölkerungszahl wieder auf rund 83,7 Mio. Personen zurück und liegt damit etwas höher als im Jahr 2020.

### Arbeitsnachfrage nach Wirtschaftszweigen und Berufen

Die Arbeitsnachfrage $avba$ in Stunden und nach Wirtschaftszweigen ist durch die Produktion und den Reallohn bestimmt. Während grundsätzlich eine steigende Realproduktion $y$ positiv auf die Arbeitsnachfrage wirkt, dämpft ein steigender Reallohn $w/p$ den Bedarf. Die Arbeitsnachfrage in Köpfen ergibt sich definitorisch durch die Division des geschätzten Arbeitsvolumens mit der exogen gesetzten Jahresarbeitszeit.

2 $$\mathrm{avba}_{i}=\beta _{1}+\beta _{2}\cdot y_{i}+\beta _{3}\cdot \frac{w_{i}}{{p}_{i}}$$

Dabei stellt *i* = 1, …, 63 den Index für die Wirtschaftszweige dar. Die Arbeitsvolumenquoten $\textit{avbaq}$ – definiert als Anteil der Arbeitsstunden einer Berufsgruppe im Wirtschaftszweig – hängen allein von den entsprechenden Lohndifferenzen ab (Maier et al. 2014). Das heißt, die Arbeitsvolumenquote sinkt, wenn der Berufslohn in den 144 Berufsgruppen *o* im Wirtschaftszweig schneller steigt als der Durchschnittslohn im Wirtschaftszweig insgesamt.

3 $$\text{avbaq}_{i,o}=\beta _{1}+\beta _{2}\cdot \frac{w_{i,o}}{w_{i}}$$

In Kombination ergibt die Schätzung des wirtschaftszweigspezifischen Arbeitsvolumens und die Arbeitsvolumenquoten nach Berufen im Wirtschaftszweig das Arbeitsvolumen $av$ nach Wirtschaftszweigen und Berufen in Stunden.

4 $$av_{i,o}=\text{avbaq}_{i,o}\cdot \mathrm{avba}_{i}$$

Die Zahl der Erwerbstätigen $e$ nach Wirtschaftszweig und Beruf ergibt sich per Definition durch Division der Arbeitsvolumen mit der Jahresarbeitszeit. Dabei wird die Dynamik der exogen vorgegebenen Jahresarbeitszeit nach Wirtschaftszweigen auf alle Berufsgruppen übertragen.

Die wirtschaftszweigspezifischen Berufslöhne wiederum, die in die Schätzgleichung für die Arbeitsvolumenquoten bestimmend einfließen, ergeben sich aus der Lohnentwicklung in der jeweiligen Berufsgruppe $w_{o}$ und stehen in Abhängigkeit zur wirtschaftszweigspezifischen Produktivität $y/e$. Je produktiver ein Wirtschaftszweig wird, desto höher fällt die Entlohnung aus.

5 $$w_{i,o}=\beta _{1}+\beta _{2}\cdot w_{o}+\beta _{3}\cdot \frac{y_{i}}{e_{i}}$$

Die Berufslohnentwicklung $w_{o}$ wiederum hängt neben dem Durchschnittslohn $W$ von berufsspezifischer Knappheit ab. Das heißt, der Lohn in einer Berufsgruppe nimmt entsprechend zu, je enger das Verhältnis der Erwerbstätigen $e$ zu den Erwerbspersonen $ep$ ist.

6 $$w_{o}=\beta _{1}+\beta _{2}\cdot W+\beta _{3}\cdot \frac{e_{o}}{ep_{o}}$$

Während die Anzahl der Erwerbstätigen und das Arbeitsvolumen aus der VGR stammt, geht die Information, welche Berufe in welchen Wirtschaftszweigen nachgefragt werden, aus dem Mikrozensus und der Beschäftigtenhistorik der Bundesagentur für Arbeit hervor. Die Strukturinformationen des Mikrozensus dienen dazu, die verschiedenen Datenquellen mittels iterativer Randsummenanpassungsverfahren (Bachem und Korte 1979) so aufeinander abzustimmen, dass die sozialversicherungspflichtig Beschäftigten und ausschließlich geringfügig Beschäftigten nach ausgeübtem Beruf der Beschäftigtenhistorik und die Erwerbstätigen nach Wirtschaftszeig mit den Erwerbstätigen nach VGR übereinstimmen. Die berufsspezifischen Löhne sind Tageslöhne von Vollzeitbeschäftigten aus der Beschäftigtenhistorik (Mönnig et al. 2019). Sie werden nach Schätzung der zensierten Löhne (Büttner und Rässler 2008) direkt aus der Beschäftigtenhistorik entnommen.

### Arbeitsangebot nach erlerntem Beruf

Wie bereits im vorherigen Abschnitt abgeleitet, nimmt das Arbeitsangebot auf Berufsgruppenebene Einfluss auf die Lohnentwicklung und somit auch auf die Arbeitsnachfrage im ökonomischen Kernmodell INFORGE. Jedoch wirkt sich auch die ökonomische Entwicklung auf das Arbeitsangebot aus.

Grundlage der Arbeitsangebotsprojektion ist die QuBe-Bevölkerungsprojektion nach Geschlecht, Alter und Nationalität. Über ein Kohorten-Komponentenmodel (Kalinowski et al. 2021) wird das Arbeitsangebot nach erlerntem Beruf ermittelt. Hierfür werden die Bestände von Schülern und Studierenden mit konstanten Übergangsquoten des Jahres 2018 fortgeschrieben. Die Berufswahl stützt sich auf mikrozensusbasierte Auswertungen der Abgänger aus dem beruflichen Bildungssystem nach Berufsgruppen. Erkennbare Entwicklungen in der Berufswahl innerhalb einer Qualifikationsstufe^3^ werden mit logarithmierten Trends fortgeschrieben. Die Fortschreibung des Bestandes an Personen außerhalb des Bildungssystems nach Nationalität, Berufsgruppen, Qualifikationen, Geschlecht und Alter ergibt sich durch eine einfache Gleichung: Der Bestand am Jahresende entspricht dem Vorjahresbestand zuzüglich der Nettoübergänge aus dem Bildungssystem, des Wanderungssaldos und abzüglich der Sterbefälle. Für die Bestimmung der zukünftigen Erwerbspersonen wird der Anteil der Erwerbspersonen an der Bevölkerung nach Nationalität, Alter, Qualifikation und Geschlecht mit logarithmierten auslaufenden Trends fortgeschrieben.

Die Erwerbsquoten werden differenziert nach Geschlecht, 14 Altersgruppen und Nationalität (Deutsche/Nichtdeutsche) in Abhängigkeit von autonomen Trends und arbeitsmarktrelevanten Indikatoren (preisbereinigtes Bruttoinlandsprodukt (BIP) pro Kopf, realem Stundenlohn, Erwerbstätigen pro Erwerbsperson sowie Dienstleistungsanteil (Cedefop 2010; Kriechel et al. 2013)) auf Basis des Zeitraums von 1991 bis 2017 geschätzt. Dabei werden alle Schätzansätze einzeln und in unterschiedlichen Kombinationen mit Zeittrends und/oder Dummy-Variablen getestet. Außerdem wurden die Schätzgleichungen auf lag-Strukturen untersucht. Die Schätzgleichungen sind alle logarithmiert. Entsprechen die Vorzeichen den theoretischen, aus der Literatur abgeleiteten Erwartungen einer positiven signifikanten Korrelation (t-Statistik) und es besteht keine Autokorrelation (Durbin-Watson), werden sie in das Model übernommen. In gut einem Drittel der Fälle wird ein Bestimmtheitsmaß von mindestens 80 % erreicht.

Es zeigt sich, dass die konjunkturelle Entwicklung (BIP/Kopf) vor allem bei Frauen (deutsche wie auch nicht deutsche) ein guter Indikator für die Entwicklung der Erwerbsquote ist. Auch der Dienstleistungsanteil weist insbesondere bei den über 40-Jährigen eine hohe Erklärungskraft auf. Die Erwerbstätigenknappheit liefert in Bezug auf Frauen hingegen kaum Erklärung bezüglich ihrer Erwerbsneigung. Der Zeittrend bei den jüngeren weiblichen Altersgruppen zeigt, dass sowohl bei deutschen als auch bei nichtdeutschen Frauen ein (autonomer) Trend zu einer erhöhten Erwerbsneigung besteht. Dies wird auch durch die Erkenntnis bestärkt, dass Reallohnsteigerungen kaum zu einer erhöhten Erwerbsneigung führen. Besonders stark fällt dies bei den ausländischen Frauen auf. Bei den Männern ist die Erwerbslosenquote – vor allem in den mittleren und älteren Jahrgängen – ein gutes Erklärungsmaß – die Wirtschaftsentwicklung hingegen nicht. Insbesondere in den jüngeren Jahrgängen nimmt hingegen der Reallohn positiv Einfluss auf die Erwerbsneigung. Die Erwerbsquoten $EQ$ nach Geschlecht $g$, Alter $a$ und Nationalität $n$ werden mittels eines Schätzansatzes auf die Erwerbsquoten nach Qualifikationen $q$ übertragen (Gl. 7).

7 $$EQ_{n,g,a,q}={\beta _{1}}+{\beta _{2}}\cdot EQ_{n,g,a}$$

Die Elastizität $\beta _{2}$ gibt wieder, ob die Erwerbsquote bei gleichem Geschlecht, gleicher Nationalität und gleichem Alter entsprechend stärker oder schwächer steigt (vgl. Dunn 1960). Die Wachstumsrate von $EQ_{n,g,a,q}$ wird anschließend auf die Erwerbsquoten nach erlerntem Beruf, differenziert nach 144 Berufsgruppen, übertragen.

### Berufliche Mobilität – Arbeitsangebot nach ausgeübten Berufen

Für eine Bilanzierung von Arbeitskräftebedarf und -angebot nach Berufen ist jedoch nicht das Angebot nach erlerntem Beruf ausschlaggebend, sondern jenes Arbeitsangebot, welches unter Berücksichtigung beruflicher Mobilitätsprozesse zustande kommt. Diese berufliche Mobilität wird in Mobilitätsmatrizen widergespiegelt. Sie geben die Wahrscheinlichkeit einer Erwerbstätigkeit in einem Erwerbsberuf, in Abhängigkeit vom höchsten erlernten beruflichen Abschluss wieder (Maier und Helmrich 2012). Sie liegen getrennt nach Nationalität, Geschlecht, Alter (15 bis 34 Jahre, 35 bis 49 Jahre und über 50 Jahre) und vier Qualifikationsstufen für jeweils 144 Berufsgruppen vor. Diese Matrizen sind Ergebnis vergangener Mobilitätsprozesse und spiegeln somit zum einen die Tätigkeitsmöglichkeiten in Abhängigkeit vom erlernten beruflichen Abschluss wider. Zum anderen ist das Ausmaß an beruflicher Flexibilität aber auch das Ergebnis vergangener Marktchancen und -risiken. Tab. 2 zeigt die berufliche Mobilitätsmatrix, aggregiert aus allen Bevölkerungsgruppen, des Jahres 2017 nach Berufsbereichen (Einsteller der KldB 2010). Auf der Diagonalen der Tabelle ist der Anteil der Personen eingetragen, die in ihrem erlernten Berufssegment erwerbstätig sind. Bei den „Sprach‑, Literatur‑, Geistes‑, Gesellschafts- und Wirtschaftswissenschaften, Medien, Kunst, Kultur und Gestaltung“ ist dieser Steher-Anteil mit rund 24,2 % am geringsten. Rund 25,2 % wandern in den Berufsbereich „Unternehmensorganisation, Buchhaltung, Recht und Verwaltung“ ab, weitere 21,2 % in den Bereich „Gesundheit, Soziales, Lehre und Erziehung“. Im letztgenannten Berufsbereich ist der Steheranteil mit rund 73,5 % am höchsten. Personen ohne erlernten Beruf sind mit 30,4 % am häufigsten in „Verkehr, Logistik, Schutz und Sicherheit“ erwerbstätig.

**Table 2:** 

		Berufsbereich ausgeübter Beruf									
		1	2	3	4	5	6	7	8	9	Summe
Berufsbereich erlernter Beruf		Land‑, Forst- und Tierwirtschaft und Gartenbau	Rohstoffgewinnung, Produktion und Fertigung	Bau, Architektur, Vermessung und Gebäudetechnik	Naturwissenschaft, Geografie und Informatik	Verkehr, Logistik, Schutz und Sicherheit	Kaufm. Dienstl., Warenhandel, Vertrieb, Hotel und Tourismus	Unternehmensorg., Buchhaltung, Recht u. Verwaltung	Gesundheit, Soziales, Lehre und Erziehung	Sprach‑, Literatur‑, Geistes‑, Gesellschafts- u. Wirtschaftswiss	
1	Land‑, Forst- und Tierwirtschaft und Gartenbau	*42,9*	9,1	4,8	1,2	15,7	8,8	9,4	6,9	1,2	100
2	Rohstoffgewinnung, Produktion und Fertigung	1,4	*50,5*	6,1	2,9	15,2	8,6	8,6	4,8	2,0	100
3	Bau, Architektur, Vermessung und Gebäudetechnik	1,7	15,5	*48,7*	1,4	16,2	5,5	6,8	3,2	1,2	100
4	Naturwissenschaft, Geografie und Informatik	0,9	13,5	1,5	*38,5*	5,7	7,0	16,5	13,1	3,3	100
5	Verkehr, Logistik, Schutz und Sicherheit	0,8	8,4	3,2	1,0	*64,2*	6,7	11,0	3,7	1,1	100
6	Kaufmännische Dienstleistungen, Warenhandel, Vertrieb, Hotel und Tourismus	0,9	6,6	1,2	1,1	13,0	*49,7*	18,2	7,0	2,2	100
7	Unternehmensorganisation, Buchhaltung, Recht und Verwaltung	0,7	3,9	0,8	1,8	6,5	11,3	*66,2*	5,5	3,4	100
8	Gesundheit, Soziales, Lehre und Erziehung	0,8	3,2	0,5	0,6	5,3	6,1	8,5	*73,5*	1,5	100
9	Sprach‑, Literatur‑, Geistes‑, Gesellschafts- und Wirtschaftswiss., Medien, Kunst, Kultur u. Gestaltung	0,5	7,4	1,4	2,3	6,0	11,5	25,2	21,4	*24,2*	100
Ohne erlernten Beruf		2,6	21,7	6,8	1,1	30,4	16,0	9,2	10,9	1,3	100
Summe		2,2	18,9	6,1	3,1	14,7	13,0	20,1	18,9	2,9	100

Quelle: Forschungsdatenzentrum der Statistischen Ämter des Bundes und der Länder, Mikrozensus 2017, eigene Berechnungen; nur Erwerbstätige ohne Personen in Bildung und ohne Militärberufe

Für die Projektion wird unterstellt, dass die beobachteten Mobilitäten auch für die Zukunft Bestand haben. Mit einer langfristigen Veränderung des Arbeitsangebotes nach den genannten Bevölkerungsgruppen verändert sich somit auch das entsprechende Arbeitsangebot für die mit den jeweiligen erlernten Berufen ausübbaren Tätigkeiten. Um die Veränderung von Marktchancen gleichermaßen zu berücksichtigen, wird auch die Beziehung zwischen der berufsspezifischen Lohnentwicklung und der Veränderung des Steher-Anteils modelliert (Maier et al. 2017). So wird für den Zeitraum von 2005 bis 2017 geprüft, ob der Steher-Anteil $\text{stayer}_{o}$ in einer Berufsgruppe auf Veränderungen der berufsspezifischen Entlohnungsmöglichkeiten reagiert hat (Gl. 8).

8 $$\text{stayer}_{o}=\delta _{1}+\delta _{2}\frac{w_{o}}{w_{o}^{\mathrm{ref}}},\mathrm{mit}o=144\,\text{Berufsgruppen}$$

Dabei stellt $w_{o}$ den Lohn in der Berufsgruppe dar und $w_{o}^{\mathrm{ref}}$ gibt den Referenzlohn wieder. Dies ist der Durchschnittlohn, den eine Person mit ihrer beruflichen Qualifizierung erhalten kann. Er berechnet sich aus den Durchschnittslöhnen aller Berufsgruppen, gewichtet mit den entsprechenden Mobilitätsanteilen. In Summe kann in 74 von 144 Berufsgruppen zwischen 2005 und 2017 nachgewiesen werden, dass sich mit einer relativen Erhöhung/Verringerung des Berufslohns auch der Anteil der Steher erhöht/verringert hat. Für zehn weitere Berufsordnungen wird die Elastizität der übergeordneten Berufshauptgruppen auf die darunterliegenden Berufsgruppen übertragen. Für diese Berufe wird auch in Zukunft unterstellt, dass eine Anpassungsreaktion des Arbeitsangebotes stattfindet, wenn sich die berufsspezifischen Löhne (siehe Gl. 5) verändern. Damit ergibt sich bei einer veränderten Nachfrage nach Berufen auch ein verändertes Angebot an Erwerbspersonen, die für die jeweiligen Berufe zur Verfügung stehen, was wiederum Rückwirkungen auf den berufsspezifischen Lohn (siehe Gl. 6) hat.

## Auswirkungen der COVID-19-Pandemie

Um die Anpassungsprozesse von QINFORGE zu illustrieren, vergleichen wir ein kontrafaktisches Szenario, welches ausgehend vom Jahr 2019 die empirisch erfassten Trends und Verhaltensweisen in die Zukunft projiziert (Maier et al. 2020), mit einer Basisprojektion, welche den durch die Corona-Pandemie bedingten Wirtschaftseinbruch (StBA 2020) abbildet. Tab. 3 zeigt in einem Überblick die Unterschiede zwischen einer Welt ohne Corona-Krise und der QuBe-Basisprojektion, welche die vermuteten Auswirkungen der Corona-Pandemie in der kurzen und mittleren Frist berücksichtigt. Die Basisprojektion stützt sich dabei auf Erkenntnisse von Umfrage- und Registerdaten mit Stand September 2020. Nicht in Tab. 3 aufgeführt, aber Teil der Basisprojektion sind die stützenden Maßnahmen in Höhe von 130 Mrd. €, die durch das Konjunkturpaket der Bundesregierung am 3. Juni 2020 aktiviert wurden. Für die Vielzahl der hierfür notwendigen Annahmen sei auf Wolter et al. (2020) verwiesen. Die Unterschiede zwischen dem kontrafaktischen Szenario und der Qube-Basisprojektion werden im folgenden Abschnitt aufgezeigt.

**Table 3:** 

Exporte	Rückgang über alle Wirtschaftszweige wegen einbrechendem Welthandel
Importe	Weniger Importe für Pharma- und Textilindustrie, da mehr im Inland hergestellt wird
Investitionen	Investitionsattentismus aufgrund von Unsicherheit
Gesundheitsausgaben	Steigende Ausgaben wegen Pandemie
Konsum der privaten Haushalte	Weniger Nachfrage aufgrund des Shutdowns vor allem bei Verwendungszwecken wie „Möbel, Innenausstattung, Teppiche u. ä.“, „Pauschalreisen“, „Verpflegungsdienstleistungen“, „Beherbergungsdienstleistungen“ und „Freizeit und Kulturdienstleistungen“ Stärkere Nachfrage nach dem Verwendungszweck „Telefon- und Telefaxdienstleistungen, Internet“
Arbeitsvolumen	Stundenreduktion für das Gastgewerbe
Bestand an Personenkraftwagen	Rückgang
Ölpreis	Rückgang
Jahresarbeitszeit	Verringerung der Jahresarbeitszeit aufgrund geringerer Arbeitskräfteknappheit

## Ergebnisse

### Ökonomische Entwicklung

Tab. 4 enthält die Kenngrößen der ökonomischen Entwicklung und des Arbeitsmarktes der QuBe-Basisprojektion sowie die Differenz der Größen zum kontrafaktischen Szenario. Ohne die Einschränkungen der Corona-Pandemie wäre für das Jahr 2020 ein Wachstum des preisbereinigten Bruttoinlandsproduktes (BIP) in Höhe von 1,5 % möglich gewesen. Die durchschnittliche Wachstumsrate von 2020 bis 2040 hätte rund 0,7 % betragen. In der Basisprojektion wird im Jahr 2020 aufgrund des „Shutdowns“ und dem damit verbundenen Wirtschaftseinbruch ein Rückgang des preisbereinigten BIP im Vergleich zum Jahr 2019 in Höhe von 7,0 % errechnet.^4^ Dies ist auf die geringere Investitionsneigung, den schrumpfenden Welthandel mit geringeren Exporten aus Deutschland, Produktionsstopps und zerrissene Lieferketten zurückzuführen. Die Schließungen zur Reduktion von Infektionsketten haben zudem stark negative Wirkungen auf den Konsum der privaten Haushalte vor allem in den Bereichen Freizeit, Tourismus, Kultur und Sport. Ausgebliebene Reisetätigkeiten und die geringeren Konsummöglichkeiten aufgrund geringeren Einkommens (Kurzarbeit) tragen überdies zum Rückgang bei. Die höheren Konsumausgaben des Staates in der Basisprojektion im Vergleich zum kontrafaktischen Szenario zeigen bereits die Folgen des Konjunkturpaketes (Wolter et al. 2020).

**Table 4:** 

	QuBe-Basisprojektion								Differenz Basisprojektion – kontrafaktisches Szenario (ohne COVID-19-Pandemie)							
	2020	2021	2022	2023	2025	2030	2035	2040	2020	2021	2022	2023	2025	2030	2035	2040
BIP in Mrd. Euro (preisbereinigt, Basis 2015)	3014	3191	3302	3351	3361	3468	3615	3742	−275	−129	−48	−21	−49	−61	−68	−45
BIP pro Kopf (preisbereinigt, Basis 2015)	36170	38219	39474	40001	40020	41229	43047	44683	−3303	−1548	−576	−248	−584	−724	−805	−535
Konsum des Staates in Mrd. Euro (preisbereinigt, Basis 2015)	669	681	690	696	703	734	770	796	3	8	9	7	−2	−3	−3	−2
Konsum privater Haushalte in Mrd. Euro (preisbereinigt, Basis 2015)	1505	1594	1650	1698	1733	1838	1956	2043	−144	−71	−32	−2	−4	−6	−9	−6
Bauinvestitionen in Mrd. Euro (preisbereinigt, Basis 2015)	327	340	334	332	326	315	314	311	−4	5	−4	−3	−4	−3	−3	−2
Ausrüstungsinvestitionen in Mrd. Euro (preisbereinigt, Basis 2015)	364	421	436	453	455	476	513	552	−56	−7	2	15	8	2	0	3
Exporte (preisbereinigt, Basis 2015)	1386	1467	1595	1633	1690	1893	2113	2385	−197	−151	−61	−59	−69	−63	−75	−47
Importe (preisbereinigt, Basis 2015)	1230	1317	1430	1480	1556	1796	2056	2347	−142	−95	−23	−15	−22	−11	−22	−7
Erwerbsbevölkerung in Mio. Personen^a^	59	59	58	58	58	57	55	53	0	0	0	0	0	0	0	0
Erwerbspersonen in Mio. Personen	47	47	47	47	46	45	45	45	0	0	0	0	0	0	0	0
Erwerbstätige in Mio. Personen	45	45	46	46	45	44	44	44	−1	−1	0	0	0	0	0	0
Erwerbstätigenquote in Prozent^b^	77	77	78	78	78	78	80	82	−1	−1	0	0	0	0	0	0
Erwerbslose in Mio. Personen	2	1	1	1	1	1	1	1	0	0	0	0	0	0	0	0
Arbeitsvolumen in Mrd. Stunden	60	63	64	64	63	62	62	61	−4	−1	0	0	0	0	0	0
Arbeitsvolumenpotenzial in Mrd. Stunden	70	70	70	70	69	68	67	67	0	0	0	0	0	0	0	0
Entwicklung des Stundenlohnes (Basis 2015)^c^	113	114	117	122	129	147	166	183	−1	−2	−2	−1	0	0	0	0
Jahresarbeitszeit der Arbeitnehmer in Stunden	1270	1334	1338	1333	1334	1337	1341	1344	−70	−2	3	−2	−3	−4	−5	−5

Quelle: Forschungsdatenzentrum der Statistischen Ämter des Bundes und der Länder, Mikrozensen 1997–2017 und Volkswirtschaftliche Gesamtrechnungen des Statistischen Bundesamts; Beschäftigtenhistorik der Bundesagentur für Arbeit; alle Werte geschätzte Werte, QuBe-Projekt, sechste Welle

^a^Bevölkerung im Alter von 15 bis unter 70 Jahre

^b^Erwerbstätige bezogen auf die erwerbsfähige Bevölkerung

^c^Stundenlohn ist nicht preisbereinigt

Die Erholungsphase des Exports erstreckt sich entsprechend den Modellrechnungen aufgrund der weltweiten Unsicherheiten und unterschiedlichen Betroffenheit durch das Corona-Virus hingegen auf rund zwei Jahre („langes V“). Unabhängig von der Corona-Pandemie sind die Risiken im Außenhandel – vor allem aufgrund der schwer einzuschätzenden Handelspolitik der USA – deutlich gestiegen. Ab dem Jahr 2025 ähneln die Wachstumsraten der Basisprojektion den erwarteten Wachstumsraten ohne Corona-Pandemie (kontrafaktisches Szenario). Der Wohlstand, der ohne die derzeitige Krise womöglich erreicht worden wäre, wird jedoch voraussichtlich erst mit einer ca. dreijährigen Verzögerung erreicht.

### Entwicklung des Arbeitsmarktes

Am Arbeitsmarkt zeigt sich in der Basisprojektion vor allem ein starker Rückgang des Arbeitsvolumens in Höhe von 5,5 % im Jahr 2020.^5^ Im Jahr 2021 ist das Arbeitsvolumen allerdings fast wieder auf der Höhe des Jahres 2019. Trotz der möglichen Kurzarbeit wird aufgrund der Corona-Pandemie auch die Erwerbslosigkeit ansteigen. Selbst wenn betriebsbedingte Kündigungen oftmals vermieden werden können, so werden freiwerdende Stellen aufgrund der Unsicherheiten zunächst nicht wiederbesetzt (Hutter und Weber 2020). In den Jahren 2020 und 2021 werden jeweils rund 600.000 Erwerbstätige weniger benötigt als dies im kontrafaktischen Szenario der Fall gewesen wäre. Da sich aufgrund der schlechteren Arbeitsmarktlage auch einige Erwerbspersonen vom Arbeitsmarkt zurückziehen werden (siehe Abschn. 2.5), steigt die Zahl der Erwerbslosen in beiden Jahren mit rund 400.000 Personen im Vergleich zum kontrafaktischen Szenario geringer an.^6^

Die zunehmende Unterbeschäftigung ist in der Entwicklung des Stundenlohns und der Jahresarbeitszeit der Beschäftigten berücksichtigt: So hemmt die Corona-Pandemie vor allem in der kurzen und mittleren Frist das Lohnwachstum, welches in einem durch Fachkräfteengpässe gekennzeichneten Arbeitsmarkt im kontrafaktischen Szenario möglich gewesen wäre. In einer „Welt ohne Corona-Virus“ hätten die Arbeitnehmer auch mehr Arbeitsstunden anbieten müssen, um die Arbeitsnachfrage befriedigen zu können. In der Basisprojektion ist eine Steigerung der Jahresarbeitszeiten zwar ebenfalls notwendig, jedoch in einer etwas geringeren Dynamik. Das geringere Lohnwachstum sowie die geringeren durchschnittlichen Arbeitsstunden führen dazu, dass in der Basisprojektion zum Projektionsende im Jahr 2040 genauso viele Personen in Erwerbstätigkeit sein werden, wie es im kontrafaktischen Szenario der Fall gewesen wäre.

### Berufsspezifische Auswirkungen

Tab. 5 stellt für die Jahre von 2020 bis 2040 die Berufsgruppen (Dreisteller der KldB 2010) dar, welche im Vergleich der Basisprojektion zum kontrafaktischen Szenario die höchsten prozentualen Abweichungen in der Erwerbstätigkeit aufweisen. Da die Erwerbstätigkeit in den meisten Berufsgruppen aufgrund der COVID-19-Pandemie zurückgeht, sind die 20 Berufsgruppen mit den höchsten Erwerbstätigenverlusten dargestellt – hingegen nur 10 Berufsgruppen mit dem höchsten Zuwachs im Bedarf an Erwerbstätigen. Die Sortierung erfolgt anhand des Jahres 2020. Dabei werden nur Berufsgruppen betrachtet, die auch mindestens 20.000 Erwerbstätige stellen.

**Table 5:** 

Berufsgruppe		Jahr							
		2020	2021	2022	2023	2025	2030	2035	2040
633	Gastronomie	−12	−11	−5	−1	−2	−2	−1	0
632	Hotellerie	−11	−11	−5	−1	−2	−2	−1	0
631	Tourismus und Sport	−10	−10	−4	−1	0	0	0	0
293	Speisenzubereitung	−10	−9	−4	−1	−1	−2	0	0
941	Musik‑, Gesangs- und Dirigententätigkeiten	−8	−7	−3	0	0	−1	−1	−1
942	Schauspiel, Tanz und Bewegungskunst	−7	−6	−3	0	0	0	0	0
933	Kunsthandwerk und bildende Kunst	−7	−6	−3	0	0	−1	−1	0
514	Servicekräfte im Personenverkehr	−6	−4	−2	−1	−1	−1	−1	−1
943	Moderation und Unterhaltung	−6	−5	−3	0	0	0	0	0
845	Fahr- und Sportunterricht an außerschul. Bildungseinrichtungen	−4	−1	0	1	1	0	0	0
945	Veranstaltungs‑, Kamera- und Tontechnik	−4	−4	−3	−2	−1	−1	−1	−1
844	Lehrtätigkeit an außerschulischen Bildungseinrichtungen	−4	−1	0	1	1	0	−1	−1
944	Theater‑, Film- und Fernsehproduktion	−3	−5	−3	−2	−2	−1	0	0
634	Veranstaltungsservice und -management	−3	−4	−2	−1	−1	−1	0	0
623	Verkauf von Lebensmitteln	−3	−3	−1	0	0	0	0	0
733	Medien‑, Dokumentations- und Informationsd.	−3	−2	−1	1	0	0	0	0
511	Techn. Betrieb des Eisenbahn‑, Luft- und Schiffsverkehrs	−3	−2	−1	0	−1	−1	−1	−1
924	Redaktion und Journalismus	−3	−3	−2	−1	−1	−1	0	−1
292	Lebensmittel- und Genussmittelherstellung	−3	−2	0	0	0	0	0	0
721	Versicherungs- und Finanzdienstleistungen	−2	−2	−1	−1	−1	−1	−1	−1
…									
112	Tierwirtschaft	1	1	1	1	0	0	0	1
832	Hauswirtschaft und Verbraucherberatung	1	1	1	1	0	0	1	1
843	Lehr- und Forschungstätigkeit an Hochschulen	1	2	3	4	5	4	4	4
816	Psychologie und nicht ärztliche Psychotherapie	2	2	2	2	0	0	0	0
812	Medizinisches Laboratorium	2	2	2	2	0	0	0	0
817	Nicht ärztliche Therapie und Heilkunde	2	2	2	2	0	0	0	0
811	Arzt- und Praxishilfe	2	2	2	2	1	0	0	0
814	Human- und Zahnmedizin	2	2	3	2	1	0	0	0
813	Gesundheits- und Krankenpflege, Rettungsd. u. Geburtshilfe	3	1	2	2	0	0	0	0
821	Altenpflege	5	0	0	0	0	0	0	0

Quelle: Forschungsdatenzentrum der Statistischen Ämter des Bundes und der Länder, Mikrozensen 1997–2017 und Volkswirtschaftliche Gesamtrechnungen des Statistischen Bundesamts; Beschäftigtenhistorik der Bundesagentur für Arbeit; Berechnung und Darstellung QuBe-Projekt, sechste Welle; nur Berufsgruppen mit mindestens 20.000 Erwerbstätigen

Für den Bedarf an Erwerbstätigen in den Berufsgruppen ist die Arbeitsnachfrage in Stunden in den jeweiligen Branchen ausschlaggebend. Die Berufsgruppen sind deshalb entsprechend ihrer Verteilung auf die Branchen betroffen, auch wenn ihre explizite Tätigkeit vielleicht mehr oder weniger gefragt ist. Am stärksten ist der Rückgang im jeweils zweistelligen Ausmaß in den Berufsgruppen „Gastronomie“, „Hotellerie“, „Tourismus und Sport“ und „Speisenzubereitung“. Zuwächse erwartet das Modell in den Berufen des Gesundheitsbereichs sowie in „Lehr- und Forschungstätigkeiten an Hochschulen“. Dies ist eine Folge des Konjunkturpaketes, welches eine mittelfristige staatliche Förderung für das Bildungswesen sowie für Forschung und Entwicklung vorsieht. In allen anderen in Tab. 5 genannten Berufsgruppen nähert sich die Erwerbstätigkeit bis zum Jahr 2025 wieder dem Niveau der Basisprojektion an.

Tab. 6 zeigt, dass in den Berufsgruppen mit einer zurückgehenden Erwerbstätigkeit auch ein geringeres Lohnwachstum zu erwarten ist, als es ohne die COVID-19-Pandemie der Fall gewesen wäre. Allerdings zeigt sich ebenfalls bis zum Jahr 2025 ein Aufholeffekt. Für die Berufe des Gesundheitsbereichs erwartet das Modell ein höheres Lohnniveau im Jahr 2020 als ohne Einfluss der Krise. Allerdings führen die Budgetrestriktionen im Gesundheitsbereich auch dazu, dass es sich bei den Lohnsteigerungen lediglich um einen Vorzieheffekt handelt. Ab dem Jahr 2023 wird in QINFORGE in der Basisprojektion für diese Berufsgruppen dasselbe nominale Lohnniveau prognostiziert wie im kontrafaktischen Szenario. Lediglich in „Lehr- und Forschungstätigkeiten an Hochschulen“ zeigt sich in der Basisprojektion langfristig ein höheres Lohnniveau.

**Table 6:** 

Berufsgruppe		Jahr							
		2020	2021	2022	2023	2025	2030	2035	2040
633	Gastronomie	−7	−7	−2	1	1	1	0	0
632	Hotellerie	−4	−5	−2	0	1	1	1	0
631	Tourismus und Sport	−5	−7	−3	−1	1	0	0	0
293	Speisenzubereitung	−6	−6	−2	1	1	1	0	0
941	Musik‑, Gesangs- und Dirigententätigkeiten	−5	−6	−3	0	1	0	0	0
942	Schauspiel, Tanz und Bewegungskunst	−3	−4	−2	0	1	0	0	0
933	Kunsthandwerk und bildende Kunst	−5	−7	−4	0	1	0	0	0
514	Servicekräfte im Personenverkehr	−2	−2	−1	0	0	0	0	0
943	Moderation und Unterhaltung	−3	−4	−2	0	1	0	0	0
845	Fahr- und Sportunterricht an außerschul. Bildungseinricht	−2	−3	−2	0	1	0	0	0
945	Veranstaltungs‑, Kamera- und Tontechnik	−1	−3	−2	−1	0	0	0	0
844	Lehrtätigkeit an außerschulischen Bildungseinrichtungen	−2	−2	−2	0	1	0	0	0
944	Theater‑, Film- und Fernsehproduktion	−2	−5	−4	−2	0	0	0	0
634	Veranstaltungsservice und -management	−2	−4	−3	−1	0	0	0	0
623	Verkauf von Lebensmitteln	−1	−4	−3	−1	1	0	0	0
733	Medien‑, Dokumentations- und Informationsd.	−1	−2	−2	0	0	0	0	0
511	Techn. Betrieb des Eisenbahn‑, Luft- und Schiffsverkehrs	−1	−2	−1	0	0	0	0	0
924	Redaktion und Journalismus	0	−1	0	0	0	0	0	0
292	Lebensmittel- und Genussmittelherstellung	−1	−3	−2	−1	0	0	0	0
721	Versicherungs- und Finanzdienstleistungen	−1	−3	−3	−1	0	0	0	0
…									
112	Tierwirtschaft	1	−2	−3	−1	0	0	0	0
832	Hauswirtschaft und Verbraucherberatung	1	−1	−2	−1	0	0	0	0
843	Lehr- und Forschungstätigkeit an Hochschulen	1	−1	0	2	3	3	2	2
816	Psychologie und nicht ärztliche Psychotherapie	1	−1	0	0	0	0	0	0
812	Medizinisches Laboratorium	2	−1	−1	0	1	0	0	0
817	Nicht ärztliche Therapie und Heilkunde	2	−2	−1	0	1	0	0	0
811	Arzt- und Praxishilfe	3	−2	−2	0	0	0	0	0
814	Human- und Zahnmedizin	1	−1	0	0	1	0	0	0
813	Gesundheits- und Krankenpflege, Rettungsd. u. Geburtshilfe	2	−2	−2	0	0	0	−1	−1
821	Altenpflege	3	−3	−3	−1	0	0	0	0

Quelle: Forschungsdatenzentrum der Statistischen Ämter des Bundes und der Länder, Mikrozensen 1997–2017 und Volkswirtschaftliche Gesamtrechnungen des Statistischen Bundesamts; Beschäftigtenhistorik der Bundesagentur für Arbeit; Berechnung und Darstellung QuBe-Projekt, sechste Welle; nur Berufsgruppen mit mindestens 20.000 Erwerbstätigen

Für die Veränderung des Arbeitsangebotes nach Berufen sind zwei Größen entscheidend (vgl. Abschn. 2.5 und 2.6): zum einen die Erwerbsquoten, die sich aufgrund der unterschiedlichen Konjunktur in beiden Szenarien voneinander unterscheiden. Zum anderen die berufliche Flexibilität der Erwerbspersonen. So reagieren die Erwerbspersonen auf die schlechteren Erwerbschancen in ihren erlernten Berufen und bieten ihre Arbeitskraft stattdessen in anderen, tätigkeitsähnlichen Berufen an.

Tab. 8 im Anhang macht deutlich, dass das Arbeitsangebot vor allem in Berufsgruppen, welche ein mittleres Qualifikationsniveau voraussetzen und vorwiegend von Frauen gewählt werden, in der kurzen Frist zurückgehen könnte. Dies ist ein Ergebnis der langfristigen Trendschätzungen, wo sich ein signifikant positiver Zusammenhang zwischen der Frauenerwerbsquote und der Konjunktur (BIP/Kopf) offenbarte. In den Berufsgruppen „Reinigung“, „Gastronomie“ und „Hotellerie“ zeigt sich verbunden mit berufsspezifischen Abwanderungen sogar langfristig ein geringeres Arbeitsangebot. Von der Krise profitieren können die vorwiegend männlich dominierten Bauberufe. Im Rahmen ihrer beruflichen Mobilität bieten hier Erwerbspersonen aus der Industrie, welche im Zuge der COVID-19-Krise Arbeitsplätze abbaut, ihre Arbeitskraft verstärkt an.

Tab. 7 zeigt mit der Gegenüberstellung der „Erwerbslosen“ nach Berufsgruppen zwischen beiden Szenarien (Basisprojektion und kontrafaktisches Szenario) das Ergebnis der bedarfs- und angebotsseitigen Anpassungsprozesse. Bei der Zahl der Erwerbslosen handelt es sich um die Differenz zwischen den Erwerbspersonen, die ihre Arbeitskraft in einer Berufsgruppe anbieten und dem Bedarf an Erwerbstätigen in dieser Berufsgruppe. Dabei bleibt unberücksichtigt, dass die Erwerbspersonen ihre Arbeitskraft in der Praxis auch in mehreren Berufen anbieten können.

**Table 7:** 

Berufsgruppe		Jahr							
		2020	2021	2022	2023	2025	2030	2035	2040
633	Gastronomie	130	94	3	−44	−23	−15	−6	−4
293	Speisenzubereitung	69	46	−2	−25	−11	−8	−4	−3
513	Lagerwirtschaft, Post und Zustellung, Güterumschlag	28	23	19	4	0	−5	−3	−4
632	Hotellerie	28	23	5	−6	−3	−6	−5	−5
251	Maschinenbau- und Betriebstechnik	18	13	4	−3	−1	−3	−2	−2
521	Fahrzeugführung im Straßenverkehr	18	28	18	−1	−4	−3	−2	−3
721	Versicherungs- und Finanzdienstleistungen	16	10	10	2	1	2	4	3
242	Metallbearbeitung	14	9	3	−1	0	−1	−1	−2
621	Verkauf (ohne Produktspezialisierung)	14	14	12	−1	−5	−5	−2	1
713	Unternehmensorganisation und -strategie	13	7	9	−2	−3	−2	1	0
631	Tourismus und Sport	12	11	3	−1	−1	−1	−1	0
623	Verkauf von Lebensmitteln	11	8	2	−2	−1	−2	−1	0
292	Lebensmittel- und Genussmittelherstellung	10	7	1	−2	−1	−1	−1	0
711	Geschäftsführung und Vorstand	10	8	5	−1	−1	−2	−1	−1
531	Objekt‑, Personen‑, Brandschutz, Arbeitssicherheit	10	12	9	2	0	−3	−3	−4
252	Fahrzeug‑, Luft‑, Raumfahrt- und Schiffbautechnik	8	3	2	−1	−1	−1	0	0
273	Technische Produktionsplanung und -steuerung	8	6	1	−2	−1	−2	−1	−1
611	Einkauf und Vertrieb	7	6	3	−2	−3	−3	−2	−2
244	Metallbau und Schweißtechnik	7	5	2	0	0	−1	−1	−1
844	Lehrtätigkeit an außerschulischen Bildungseinrichtungen	7	0	−1	−2	−1	0	0	0
…									
843	Lehr- und Forschungstätigkeit an Hochschulen	−4	−7	−12	−17	−20	−17	−17	−15
111	Landwirtschaft	−6	−6	−2	0	1	−1	−1	0
541	Reinigung	−7	20	33	17	1	−9	−7	−5
817	Nicht ärztliche Therapie und Heilkunde	−10	−8	−8	−7	−2	−1	−2	−1
814	Human- und Zahnmedizin	−11	−11	−13	−13	−6	−4	−4	−3
832	Hauswirtschaft und Verbraucherberatung	−11	−8	0	2	4	−1	−2	−2
831	Erziehung, Sozialarbeit, Heilerziehungspflege	−11	−10	−2	1	4	−1	0	2
811	Arzt- und Praxishilfe	−25	−17	−13	−9	2	1	0	1
821	Altenpflege	−44	2	7	6	4	3	2	2
813	Gesundheits- und Krankenpflege, Rettungsdienst und Geburtshilfe	−46	−20	−16	−13	0	4	8	14

Quelle: Forschungsdatenzentrum der Statistischen Ämter des Bundes und der Länder, Mikrozensen 1997–2017 und Volkswirtschaftliche Gesamtrechnungen des Statistischen Bundesamts; Beschäftigtenhistorik der Bundesagentur für Arbeit; Berechnung und Darstellung QuBe-Projekt, sechste Welle; nur Berufsgruppen mit mindestens 20.000 Erwerbstätigen

Unter Berücksichtigung der Auswirkungen der COVID-19-Pandemie nimmt die Zahl der Erwerbslosen (in Tausend Personen) vor allem in der „Gastronomie“, der „Speisenzubereitung“, „Lagerwirtschaft, Post und Zustellung, Güterumschlag“ und „Hotellerie“ zu. Ein langfristig höheres Arbeitsangebot als Arbeitskräftebedarf zeigt sich jedoch nur in den „Versicherungs- und Finanzdienstleistungen“. In allen anderen Berufsgruppen, deren Erwerbslosenzahlen im Jahr 2020 ansteigen, ist langfristig sogar ein Rückgang im Vergleich zum kontrafaktischen Szenario erkennbar, weil auch das Arbeitsangebot leicht abnimmt. Damit trifft die Krise in der kurzen und mittleren Frist vor allem Berufe, in welchen Fachkräfte aus dem mittleren Qualifikationsbereich, aber auch Un- und Angelernte, eine Erwerbstätigkeit ausüben.

Zu den Berufsgruppen mit einer geringeren Anzahl an Erwerbslosen im Jahr 2020 zählen zum einen die Berufe des Gesundheitswesens mit einem zunehmenden Bedarf an Erwerbstätigen, aber auch die Berufsgruppen „Hauswirtschaft- und Verbraucherberatung“ oder „Reinigung“, bei denen sich ein kleiner Teil der Erwerbspersonen vom Arbeitsmarkt zurückzieht. Langfristig ergibt sich aus QINFORGE, dass in der „Altenpflege“ und in den Bereichen „Gesundheits- und Krankenpflege, Rettungsdienst und Geburtshilfe“ etwas mehr Erwerbspersonen ihre Arbeitskraft anbieten könnten, obwohl auch der Bedarf an Erwerbstätigen in der Basisprojektion geringfügig höher ausfällt als im kontrafaktischen Szenario. Dies ist darauf zurückzuführen, dass sich die Erwerbschancen in den anderen Berufen, welche qualifizierte Pflegekräfte ausüben können, in der Basisprojektion im Vergleich zu kontrafaktischen Szenario geringer sind. Ein ähnlicher Mechanismus zeigt sich in „Lehr- und Forschungstätigkeiten an Hochschulen“. Hier ist der Bedarf an Erwerbstätigen in der „Basisprojektion“ höher, die Erwerbs- und Entlohnungschancen in anderen Berufen aber weiterhin besser, wodurch die Differenz der benötigten Erwerbstätigen und potenziell zur Verfügung stehenden Erwerbspersonen im Beruf in der Basisprojektion geringer ausfällt.

## Fazit

Die Qualifikations- und Berufsprojektionen haben zum Ziel, mittel- und langfristige berufliche Passungsprobleme aufzuzeigen, um für die Arbeits- und Bildungspolitik entsprechende Handlungsfelder benennen zu können. Dieser Bericht stellt das für die Projektionen verwendete Modellsystem QINFORGE vor. Das Alleinstellungsmerkmal von QINFORGE liegt in der empirisch fundierten Modellierung beruflicher Anpassungsprozesse durch die Verwendung beruflicher Mobilitätsmatrizen (Abschn. 2). So nimmt die berufsspezifische Entlohnung Einfluss auf die nachgefragte Anzahl an Gütern und Dienstleistungen – wie auch das zur Verfügung stehende Arbeitsangebot in einem Beruf. Ebenso werden Erwerbsquoten in Abhängigkeit von der wirtschaftlichen Entwicklung modelliert.

Die wirtschaftlichen Einschnitte in Folge der COVID-19-Pandemie bieten einen Rahmen, um die Wirkung der endogen modellierten Anpassungsprozesse zu demonstrieren und entsprechende Konsequenzen der COVID-19-Pandemie auf die berufsspezifischen Arbeitsmärkte in der kurzen, mittleren und langen Frist zu verdeutlichen. Hierfür werden zwei Szenarien miteinander verglichen. Ein kontrafaktisches Szenario schreibt langfristige Verhaltensweisen und Trends ausgehend vom Jahr 2019 fort. Die Basisprojektion versucht hingegen, bereits die Konjunktur des Jahres 2020 mit abzubilden, indem eine geringere Investitionsneigung, ein zurückgehender Konsum, ein geringerer Außenhandel und eine Reduzierung der Arbeitszeiten berücksichtigt wird. Ebenso wird das am 3. Juni 2020 verabschiedete Konjunkturpaket in Höhe von 130 Mrd. € berücksichtigt (siehe Abschn. 3).

Die COVID-19-Pandemie hat Deutschland in einer robusten wirtschaftlichen Lage getroffen. Die makroökonomischen Fundamentalvariablen der Wirtschaft waren vor dem Wirtschaftseinbruch gesund und der Arbeitsmarkt stabil – mit einer Erwerbslosenquote auf historisch niedrigem Niveau. Die Ergebnisse des Szenario-Vergleichs offenbaren, dass die Krise die Wirtschaftsleistung Deutschlands langfristig um drei Jahre zurückwerfen wird. Trotz der möglichen Kurzarbeit wird aufgrund der Corona-Pandemie auch die Erwerbslosigkeit ansteigen. Für die Jahre 2020 und 2021 prognostiziert die Basisprojektion jeweils rund 600.000 Erwerbstätige weniger als im kontrafaktischen Szenario. Da sich aufgrund der schlechteren Arbeitsmarktlage aber auch einige Erwerbspersonen vom Arbeitsmarkt zurückziehen werden, steigt die Zahl der Erwerbslosen in beiden Jahren jedoch nur um rund 400.000 Personen im Vergleich zum kontrafaktischen Szenario an.

Die Unterbeschäftigung muss in der Veränderung der Jahresarbeitszeit berücksichtigt werden und beeinflusst dadurch auch die Entwicklung des Stundenlohns: So hemmt die Corona-Pandemie vor allem in der kurzen und mittleren Frist das Lohnwachstum, welches in einem durch Fachkräfteengpässe gekennzeichneten Arbeitsmarkt im kontrafaktischen Szenario möglich gewesen wäre. Dies führt dazu, dass die Basisprojektion zum Projektionsende im Jahr 2040 in etwa genauso viele Personen in Erwerbstätigkeit berechnet wie im kontrafaktischen Szenario.

Die Annahmen der Branchenentwicklungen, welche sich auf aktuelle Erhebungen und Registerdaten mit Datenstand September 2020 stützen, offenbaren, dass das Arbeitskräfteangebot und der -bedarf nach Berufen durch die Krise unterschiedlich betroffen sein wird. Da die „Shutdowns“ vorwiegend den Tourismus, die Gastronomie sowie Kunst und Kultur treffen, zeigt sich in den entsprechenden Berufen der stärkste Rückgang im Erwerbstätigenbedarf und in den Entlohnungen. So wird es in der Basisprojektion rund fünf Jahre dauern, bis eine Erwerbstätigkeit und ein nominales Lohnniveau erreicht wird, welches der Berechnung im kontrafaktischen Szenario entspricht. Bei den Erwerbsquoten wird deutlich, dass sich vor allem Frauen im mittleren Qualifikationsbereich durch die Krise vom Arbeitsmarkt zurückziehen und damit auf Einkommen verzichten.

In Berufen des Gesundheitsbereichs steigt hingegen in der kurzen Frist die Nachfrage nach Erwerbstätigen. Aufgrund der Budgetrestriktionen des Gesundheitsbereichs werden die ebenfalls kurzfristigen Lohnsteigerungen von QINFORGE modellendogen als Vorzieheffekte gedeutet. Langfristig zeigt sich in der Basisprojektion im Gesundheitsbereich – und hier vor allem bei den Pflegeberufen – ein leicht höheres Arbeitsangebot als im kontrafaktischen Szenario. Dies ist jedoch weniger auf einen Pull-Faktor der Pflege in Form einer höheren Entlohnung, sondern eher auf geringere Pull-Faktoren aus anderen Berufen zurückzuführen. Durch die COVID-19-Pandemie wird die Entlohnung in alternativen Berufen, die für Pflegefachkräfte in Frage kommen, geringer. Eine ähnliche Arbeitsmarktsituation ergibt sich auch in den Bauberufen: Da in der Basisprojektion in der Industrie mehr Arbeitsplätze verloren gehen als im kontrafaktischen Szenario, bieten die vorwiegend männlichen Erwerbspersonen ihre Arbeitskraft eher in den tätigkeitsähnlichen Bauberufen an. Damit deutet sich an, dass die COVID-19-Krise das Arbeitsangebot in Berufen erhöhen könnte, in denen bereits heute ein Fachkräfteengpass diagnostiziert wird (Statistik der BA 2019), die aber bislang für die Erwerbspersonen weniger attraktiv waren. Gleichwohl stellt das knappe Arbeitsangebot langfristig einen hemmenden Faktor für das Wirtschaftswachstum dar. Auch in der Basisprojektion wird – insbesondere in den Pflegeberufen – das Arbeitsangebot langfristig nicht ausreichen, um die Arbeitsnachfrage zu befriedigen. Neben entsprechenden Qualifizierungsmaßnahmen wird deshalb auch Zuwanderung notwendig sein, um die Zahl der Erwerbspersonen zu erhöhen.

Für die Analyse wurde die COVID-19-Krise als externer Schock betrachtet. Sie zeigt, dass vor allem Personen in Berufen, die ein mittleres Qualifikationsniveau voraussetzen, in den Entlohnungs- und Erwerbschancen in der kurzen und mittleren Frist beeinträchtigt werden. Hier könnten individuelle Qualifizierungsmaßnahmen helfen, um die Beschäftigung in weniger von der Krise betroffenen Berufen zu erhöhen. Langfristig können sie jedoch wieder im gleichen Maße am Arbeitsmarkt teilhaben wie in einem kontrafaktischen Szenario – sofern die Arbeitswelt wieder in die alten Verhaltensmuster zurückfällt. Dies muss aber nicht der Fall sein. So zeigt sich in der Krise eine weitaus stärkere Nutzung von Homeoffice und Videokonferenztools, welche auch nach der ökonomischen Bewältigung der Krise fortbestehen kann. Hier ist bekannt, dass gerade die Zugänge zu Homeoffice-Tätigkeiten nach Qualifikationsniveau ungleich verteilt sind (Mergener 2020). Die Auswirkungen einer solchen veränderten Arbeitsweise auf die beruflichen Beschäftigungs- und Entlohnungschancen müssten in weiteren Szenarien untersucht werden.

## Anhang

**Table 8:**

Berufsgruppe		Jahr							
		2020	2021	2022	2023	2025	2030	2035	2040
832	Hauswirtschaft und Verbraucherberatung	−2	−1	1	1	1	0	0	0
541	Reinigung	−1	−1	0	0	−1	−1	−1	0
624	Verkauf von drogerie- und apothekenüblichen Waren, Sanitäts- und Medizinbedarf	−1	−1	0	0	0	0	0	0
623	Verkauf von Lebensmitteln	−1	−1	−1	0	0	0	0	0
282	Textilverarbeitung	−1	−1	0	−1	−1	−2	−2	−1
811	Arzt- und Praxishilfe	−1	0	1	1	1	0	0	1
821	Altenpflege	−1	0	1	1	1	1	1	1
714	Büro und Sekretariat	−1	−1	0	0	0	0	0	0
122	Floristik	−1	−1	0	0	0	0	0	0
115	Tierpflege	−1	−1	0	0	0	−1	−1	0
621	Verkauf (ohne Produktspezialisierung)	−1	−1	0	0	0	0	0	0
633	Gastronomie	−1	−3	−5	−5	−4	−2	−1	0
632	Hotellerie	−1	−2	−3	−3	−3	−2	−2	−2
733	Medien‑, Dokumentations- und Informationsd.	−1	−1	0	0	0	0	0	0
625	Buch‑, Kunst‑, Antiquitäten- und Musikfachhandel	−1	−1	0	0	0	0	0	0
823	Körperpflege	−1	0	0	0	−1	−1	−1	−1
732	Verwaltung	−1	−1	0	0	0	0	0	0
622	Verkauf von Bekleidung, Elektronik, Kraftfahrzeugen und Hartwaren	−1	−1	0	0	0	0	0	0
112	Tierwirtschaft	−1	−1	0	1	0	0	0	1
812	Medizinisches Laboratorium	−1	−1	0	0	0	0	0	0
…									
262	Energietechnik	0	0	0	0	0	0	0	0
223	Holzbe- und -verarbeitung	0	0	0	0	0	0	0	0
211	Berg‑, Tagebau und Sprengtechnik	0	0	0	0	−1	0	0	0
342	Klempnerei, Sanitär‑, Heizungs- und Klimatechnik	0	0	0	0	0	−1	−1	0
322	Tiefbau	0	0	1	0	0	0	0	0
212	Naturstein- und Mineralaufbereitung und -verarbeitung und Baustoffherstellung	0	1	1	0	0	0	0	0
332	Maler- und Lackierer‑, Stuckateurarbeiten, Bauwerksabdichtung, Holz- und Bautenschutz	0	1	1	0	0	−1	−1	0
333	Aus- und Trockenbau, Isolierung, Zimmerei, Glaserei, Rollladen- und Jalousiebau	0	1	1	0	0	−1	−1	0
331	Bodenverlegung	0	1	1	0	0	−1	−1	0
321	Hochbau	0	1	1	1	0	−1	−1	0

Quelle: Forschungsdatenzentrum der Statistischen Ämter des Bundes und der Länder, Mikrozensen 1997–2017 und Volkswirtschaftliche Gesamtrechnungen des Statistischen Bundesamts; Beschäftigtenhistorik der Bundesagentur für Arbeit; Berechnung und Darstellungen QuBe-Projekt, sechste Welle; nur Berufsgruppen mit mindestens 20.000 Erwerbstätigen.

## Footnotes

1 Grundsätzlich können Sprachregeln eine diskriminierende Wirkung haben. Aus Gründen der Texteffizienz und des Leseflusses wird im Folgenden jedoch das generische Maskulinum verwendet. Selbstverständlich sind damit immer alle Geschlechter gemeint.

2 Input-Output-Modelle bilden als Totalmodelle im Gegensatz zu Partialmodellen, zu denen meist Mikromodelle (Peichl et al. 2010) gezählt werden, die gesamte Volkwirtschaft und nicht nur einen Teil davon ab.

3 Ohne vollqualifizierenden beruflichen Abschluss/mit beruflichem Abschluss/Aufstiegsfortbildung, Bachelorabschluss oder Fachhochschul-Diplom/Hochschulabschlüsse ohne Bachelorabschluss oder Fachhochschul-Diplom.

4 Das tatsächliche Wirtschaftswachstum fiel mit −5 % um rund zwei Prozentpunkte geringer aus. Die Wachstumsprognose des preisbereinigten BIP von 2020 auf 2021 liegt mit rund 5,9 % hingegen um rund zwei Prozentpunkte oberhalb der Prognosen anderer Institutionen (Bundesministerium für Wirtschaft und Energie 2021: 3,0 %; OECD 2020: 2,8 %; Deutsche Bundesbank 2020: 3,0 %). Ab dem Jahr 2022 ähneln die Wachstumserwartungen der Basisprojektion den anderen Projektionen. Die substanziellen Aussagen des Szenariovergleichs verändern sich durch diese abweichenden Entwicklungen nicht.

5 Tatsächlich gingen die geleisteten Arbeitsstunden um 4,8 % zurück.

6 Für das Jahr 2020 weist die Volkswirtschaftliche Gesamtrechnung eine um 474.000 Personen höhere Anzahl an Erwerbslosen aus als 2019. Die Anzahl der erwerbstätigen Inländer liegt im Jahr 2020 um 432.000 Personen unter dem Wert des Jahres 2019. Ohne COVID-19-Krise wäre jedoch auch ein weiteres Erwerbstätigenwachstum im Vergleich zu 2019 möglich gewesen.
